# Induced Types 2 and 3 Deiodinase in Non-Thyroidal Illness Syndrome and the Implications to Critical Illness-Induced Myopathy—A Prospective Cohort Study

**DOI:** 10.3390/ijms26062410

**Published:** 2025-03-07

**Authors:** André Cardoso Braun, Thaliane Carvalho Oliveira, Ludmilla C. D. Thomazini, Gustavo Argenti, Bruno Jaskulski Kotzian, Valentina Machado, João Henrique M. Conte, Carolina Zanfir, Amanda C. A. Souto, Bruna Ulian, Josi Vidart, Simone Magagnin Wajner

**Affiliations:** 1 Endocrine Division, Hospital de Clínicas de Porto Alegre, Universidade Federal do Rio Grande do Sul (UFRGS), Porto Alegre 90035-003, Rio Grande do Sul, Brazil; abraun@hcpa.edu.br (A.C.B.);; 2 Department of Internal Medicine, Universidade Federal do Rio Grande do Sul (UFRGS), Porto Alegre 90035-003, Rio Grande do Sul, Brazil

**Keywords:** thyroid hormone, type 3 deiodinase, non-thyroidal illness syndrome

## Abstract

Loss of muscle mass and strength is a common condition associated with adverse outcomes in critically ill patients. Here, we determined the correlation between non-thyroidal illness (NTIS) and molecular alterations in the muscle of critically ill individuals. We evaluated deiodinase expression, intramuscular triiodothyronine (T3) levels, and mitochondria and sarcoplasmic reticulum components. The cellular colocalization of the enzymes and its influence on myocytes and genes regulated by T3 were shown, including those of mitochondria. A prospective cohort of 96 patients. Blood and muscular samples were collected on admission to the intensive care unit (ICU), as well as clinical data and ultrasonographic measurements. Patients with NTIS showed increased oxidative stress markers associated with critical illness in muscle biopsy, such as carbonyl content and low sulfhydryl and GSH. The distribution pattern of deiodinases in muscle and its biochemical properties showed significant pathophysiological linkage between NTIS and muscle loss, as type 3-deiodinase (D3) was highly expressed in stem cells, preventing their differentiation in mature myocytes. Despite the high type 2-deiodinase (D2) expression in muscle tissue in the acute phase of critical illness, T3 was unmeasurable in the samples. In this scenario, we also demonstrated impaired expression of glucose transporters GLUT4, IRS1, and 2, which are involved in muscle illness. Here, we provide evidence that altered thyroid hormone metabolism contributes to stem cell dysfunction and further explain the mechanisms underlying critical illness-induced myopathy.

## 1. Introduction

Changes in the skeletal muscle system are of great relevance to the homeostasis of systemic physiology as it constitutes approximately 40% of the individual body mass and is involved in a significant portion of energy expenditure and the metabolism of carbohydrates and lipids [1]. In the critical illness setting, the loss of muscle mass and strength related to systemic inflammation is a common condition associated with a variety of adverse outcomes, including prolonged need for mechanical ventilation (and failures in weaning attempts), increased length of stay in intensive care unit (ICU) and hospitalization, elevated mortality rates, and a rise in hospital costs [2,3,4]. Assessing muscle strength could be challenging in ICU patients. Traditional anthropometry is highly affected by common conditions of critical illness, such as impaired nutritional state and fluid balance [5]; thus, in the past few decades, growing evidence using image protocols to assess muscle thickness and potential loss during ICU stay took place as an alternative to traditional anthropometric methods, showing correlation with strength status in this scenario [6,7,8]. The use of point of care ultrasound assessment of the thickness of quadriceps femoris muscle protocols has been studied and utilized as a reliable method to achieve quantitative and qualitative information on the quadriceps muscle condition in a reproducible manner [9,10,11,12,13].

Thyroid hormones (TH) are essential for the myogenesis, maintenance, and functionality of skeletal muscle tissue [14]. Their availability within the intracellular milieu depends on local activation or inactivation, and their entry through the plasma membrane occurs via monocarboxylate transporters 8 and 10 (MCTH10 and MCTH8) [15]. Triiodothyronine (T3) activates stem cells and myotubules, promotes mitochondrial biogenesis [14,15], among other effects. Additionally, T3 regulates the glucose transporter GLUT4 stability, leading to its translocation to the sarcolemma [16,17,18]. The thyroid hormone metabolism apparatus is dysfunctional in critical illness. These changes, known as non-thyroidal illness (NTIS), contribute to an increased risk of morbidity and mortality in ICU patients [19,20]. NTIS is characterized by low levels of T3 and free T3 (FT3) and elevated levels of reverse T3 (rT3). Deiodination is the critical step in regulating the tissue availability of thyroid hormones. The iodothyronine deiodinases—D1, D2, and D3—are a family of oxidoreductases that activate or inactivate the thyroid hormone, respectively. The thyroid hormone-activating enzyme, type 2 deiodinase (D2), also induces myogenic differentiation by regulating mitochondrial metabolism [21], which, together with the inactivating role of the type 3 deiodinase (D3) enzyme, controls the thyroid hormone homeostasis at the cellular level [22,23].

This study aims to examine the relationship between NTIS and molecular changes in the muscle of critically ill patients. We demonstrate the colocalization of enzymes involved in thyroid hormone metabolism within myocytes, their impact on fiber health, and the genes regulated by T3, including those related to mitochondrial regulation. Furthermore, we explore the potential implications of these factors on muscle loss and increased glucose resistance in this population.

## 2. Results

### 2.1. Demographic and Clinical Findings

Ninety-six patients entered the final cohort analysis on day one, of whom 12 underwent muscle biopsy on the first day. During the first week of hospitalization, twenty-two patients were discharged, ten died, there were two withdrawals of consent, and three losses to laboratory follow-up. The mean age of patients was 59.6 years, with 56% male. Sixty-seven percent of admissions were attributed to sepsis and septic shock. The mean Simplified Acute Physiology (SAPS) score was 74.5 ± 0.7, and the mean Sequential Organ Failure Assessment (SOFA) score at admission was 8.5 ± 3.53. NTIS was present in 85 patients (88%) upon admission. Among them, 43 patients had values between 30 and 59.9 ng/dL, and 42 had levels below the detection limit of the laboratory test used (30 ng/dL). Of all patients, 25 (26%) had a total T4 lower than the lower limit of the standard laboratory reference. Twenty-eight deaths were observed on the 28th day after admission (28-day mortality of 29%), with no statistical difference in T3 levels between the survivor group (median 32.5 (IQR: 30.0–46.8 ng/dL) and the non-survivor group (median 36.0 (IQR: 30.0–53.1) according to the *t*-test (*p* = 0.53) at day 1. The mean ultrasound measurements of the femoral quadriceps muscle measured at admission in the survivor and non-survivor groups were 2.17 ± 0.8 cm and 2.33 ± 0.74 cm, respectively. Demographic and clinical parameters on day one is presented in Table 1.

Upon follow-up, the median T3 level on the seventh day of hospitalization for the survivor group on day 28 (n = 44) was 45.0 (IQR: 31.3–56.5) ng/dL, whereas the median in the non-survivor group (n = 15) was 39.5 (IQR: 30.0–47.8) ng/dL, with no difference detected (Mann–Whitney test, *p* = 0.085). Data on hormonal measures, ultrasound, and nutritional status at day 7 are demonstrated in Table 2.

### 2.2. Muscle Ultrasound Measures

Regarding ultrasound measurements, 62 patients were evaluated on the first and on the seventh days of hospitalization. There was no significant variation detected by ultrasound in the thigh’s muscle thickness from analyzed patients in the first week of ICU admission, as assessed by analysis of variances of two measures, taken on the 1st and on the 7th days (*p* = 0.97). The mean cross-sectional area of the femoral quadriceps on the 7th day for survivors on the 28th day of hospitalization was 1.85 ± 0.52. For non-survivors, it was 2.1 ± 0.8, with no statistical difference between them (*t*-test, *p* = 0.165). Additionally, the variation in plasma T3 levels between the 1st and the 7th day of hospitalization showed no correlation with the variation in muscle thickness by ultrasound measures (Spearman’s coefficient *ρ* = 0.01, *p* = 0.94). Interestingly, there was a positive correlation between the variation in muscle thickness and weight gain during the first week of hospitalization, suggesting an influence of positive fluid balance on the increased cross-sectional area of the femoral quadriceps on the 7th day, as described by a Pearson correlation of 0.307 (*p* = 0.02).

### 2.3. T3 Variations During the First Week in ICU

The variation in plasma T3 concentration on the 7th day of hospitalization negatively correlated with the hospital length of stay. Reduction in thyroid hormone levels during hospitalization was associated with a more extended stay in ICU, with a moderate Spearman coefficient *ρ* = −0.305, *p* = 0.019. Patients alive on the 28th day presented increased T3 levels. Non-survivors did not exhibit a positive variation in this hormone in the first week after ICU admission (median variation for the survivor group: 4.79 ng/dL, Mann-Whitney *p* = 0.031).

### 2.4. Analysis According to T3 Level

Data are also presented according to plasma T3 concentration, with stratified groups of patients with (1) very low T3: plasma T3 < 35 ng/dL, (2) low T3: plasma T3 between 35.1 and 59.9 ng/dL, and (3) non-NTIS group with patients with plasma T3 concentration > 60 ng/dL. This stratification was relevant to identify histological patterns in biopsies. Ultrasound measures were also analyzed considering these groups, and there were no statistical differences among them in the first week of ICU stay (ANOVA among groups *p* = 0.59).

### 2.5. Muscle Biopsies

#### Redox Homeostasis

**Figure 1:**
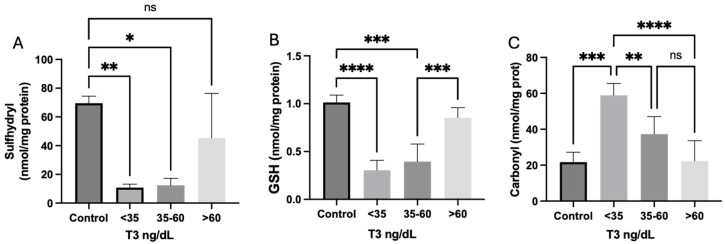
Inflammation and oxidative stress biomarkers in patients, according to their serum T3 levels. (**A**) Sulphydryl levels. (**B**) GSH levels. (**C**) Carbonyl levels. * *p* = 0.001, ** *p* = 0.0017, *** *p* = 0.002, **** *p* = 0.0001. ns: Statistically not significant.

In comparison with healthy controls, it was verified a reduced sulfhydryl and GSH and augmented carbonyl concentrations in both groups of plasmatic T3 < 35 and T3 35.1–59.9 ng/dL, suggesting a state of increased cellular oxidative stress related to inflammation among patients with NTIS, which was not observed in patients without the diagnosis, with plasma T3 concentration > 60 ng/dL. These results are shown in Figure 1.

### 2.6. Deiodinase Expression

#### 2.6.1. Type 2 Deiodinase Expression and Localization Inside the Muscle Tissue

**Figure 2:**
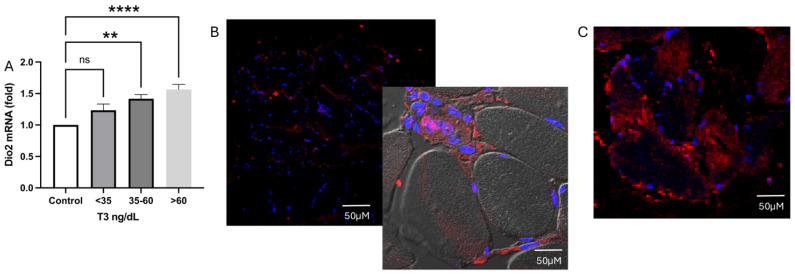
DIO2 expression and D2 protein location on muscle. DIO2 is expressed in muscle (**A**), but in those patients with serum T3 levels < 35 ng/dL, the protein is notably localized at the stem cell niche (**B**). In contrast, the protein is observed along the myocyte in patients with T3 levels > 35.1 ng/dL (**C**). ** *p* = 0.0017, **** *p* = 0.002. ns: Statistically not significant.

**Figure 3:**
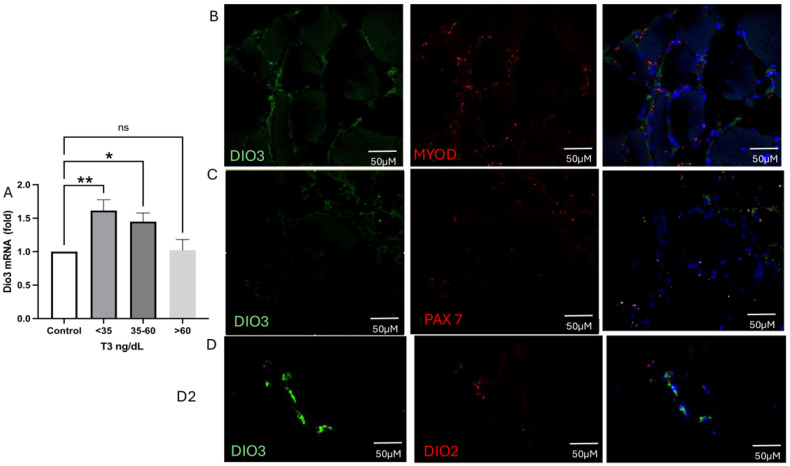
Type 3 deiodinase expression and colocalization on muscle. (**A**) DIO3 expression is augmented in the muscle of the critically ill, specifically in those with NTIS. (**B**) panel. RNAscope showing the colocalization of DIO3 and MYOD+, augmented in patients with T3 levels < 35 ng/dL. (**C**) RNAscope showing that DIO3 and PAX7+ do not colocalize in patients with T3 levels < 35 ng/dL. (**D**) RNAscope of DIO3 and DIO2, also shows that these genes do not colocalize in patients with T3 levels < 35 ng/dL. DAPI (blue), DIO3 (green), MYOD, PAX7, DIO2. (red) * *p* = 0.001, ** *p* < 0.0001. ns: Statistically not significant.

As shown in Figure 2A–C, type 2 deiodinase (DIO2) mRNA and protein synthesis were increased in the acute phase of critical illness compared to healthy controls. There is significantly more D2 protein, specifically in the stem cells of the group with plasma T3 < 35 ng/dL (Figure 2B), compared with patients with T3 levels between 35.1 and 59.9 ng/dL. In contrast, the protein is concentrated in the myocyte in patients with T3 levels > 35.1 ng/dL (Figure 3C). Interestingly, while intramuscular T3 levels in controls were 5 pmol/g, they were unmeasurable in both deficient and low serum T3 levels.

#### 2.6.2. Type 3 Deiodinase mRNA Expression and Colocalization

**Figure 4:**
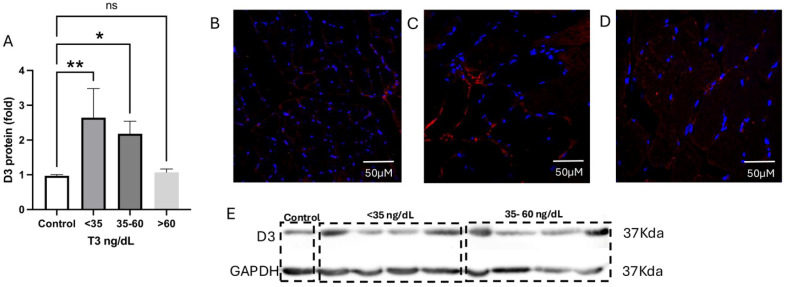
Protein levels of D3 are augmented in the muscle of critically ill patients with NTIS. (**A**,**B**) Immunofluorescence of D3 in muscle control patient. (**C**) Immunofluorescence of D3 in the muscle of a patient with T3 levels < 35 ng/dL. (**D**) Immunofluorescence of D3 in a patient’s muscle with T3 serum levels > 60 ng/dL. (**E**) The western blot shows a difference in protein levels of D3 according to the serum T3 levels. DAPI (blue), D3 (red). * *p* = 0.001, ** *p* < 0.0001. ns: Statistically not significant.

As demonstrated in Figure 3, DIO3 is highly expressed in the muscle of those patients with NTIS or T3 levels less than 60 ng/dL (Figure 3A). DIO3 mRNA colocalizes with MYOD+ (myogenic differentiation (MYOD)-positive stem cells, Figure 4B, control and Figure 3C NTIS patients) but not with PAX7+ (paired box 7 (PAX7)-positive cells, Western blot image NTIS patients). DIO3 is highly expressed in the cells of NTIS patients than in controls. Interestingly, DIO2 and DIO3 do not colocalize.

When we look at the D3 protein (Figure 4), it is possible to see that the amount of protein is augmented, especially in patients with T3 levels < 35 ng/dL (Figure 4A,C) when compared to controls and patients with T3 levels > 60 ng/dL (Figure 4A, Figure 4B, and Figure 4D, respectively).

### 2.7. Glucose Receptors and Transporter

**Figure 5:**
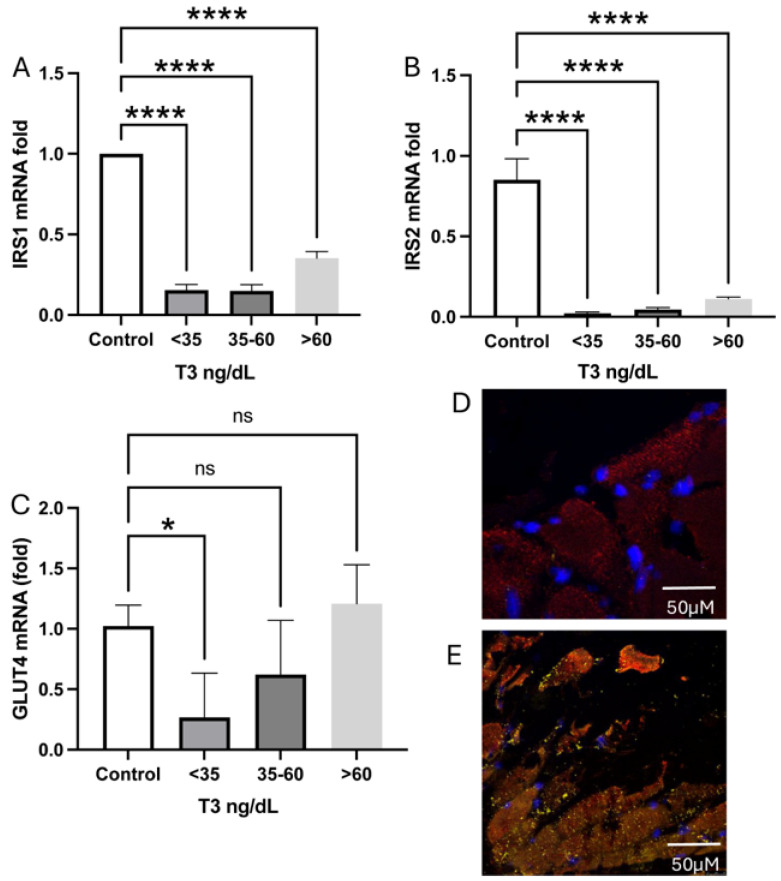
Glucose receptors in muscle cells of patients, according to their serum T3 levels. (**A**) IRS1 mRNA levels. (**B**) IRS2 mRNA levels. (**C**) GLUT4 mRNA levels. (**D**) RNAscope of MYOD+ (red) and (**E**) RNA scope of MYOD+ (red) with immunofluorescence of GLUT4 (orange) protein. DAPI (blue). * *p* = 0.002, **** *p* = 0.0001. ns: Statistically not significant.

We verified a decrease in the expression of GLUT4 mRNA in the acute phase of the disease in patients with plasmatic T3 < 35 ng/dL compared to controls. A decreased expression of IRS1 and IRS2 mRNA (Figure 5A,B) was observed. Among other biochemical pathways, these receptors are responsible for GLUT4 translocation towards the cytoplasmic membrane [24]. Strikingly, GLUT4 expression was diminished in those patients with very low T3, emphasizing the dependence of this transporter on T3 regulation. We then sought to verify whether this molecule colocalizes or not with DIO3 and observed that GLUT4 is present at the MYOD+ cells but not in the PAX7+, so colocalizing with D3.

### 2.8. Mitochondrial Dynamics and Biogenesis

**Figure 6:**
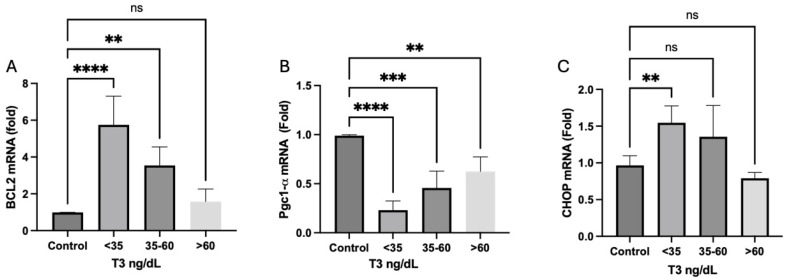
Oxidative stress and apoptosis. (**A**) Cytoprotective BCL-2 mRNA is augmented in patients with NTIS. (**B**) Antioxidant defense factor Pgc1-α mRNA is augmented in critically ill patients with or without NTIS. (**C**) Proapoptotic gene CHOP is significantly more expressed in serum T3 < 35 ng/dL groups than in controls. ** *p* = 0.0017, *** *p* = 0.002, **** *p* = 0.0001. ns: Statistically not significant.

Cytoprotective Bcl-2 proteins prevent mitochondrial permeability transition pore opening and the release of apoptogenic proteins from mitochondria, thus regulating cellular apoptosis [24]. Compared to controls, we observed a significant increase in Bcl-2 mRNA expression in the groups of patients with NTIS (plasmatic T3 < 60 ng/dL, Figure 6A). Transcriptional co-activator peroxisome proliferator-activated receptor gamma coactivator 1 alpha (Pgc1α, Figure 6B), which is a regulator of mitochondrial biogenesis and the antioxidant defense [14,24], had a reduced expression in all groups in comparison with healthy controls. Finally, our study reveals a significantly higher mRNA expression of the proapoptotic gene CHOP in patients with plasma T3 < 35 ng/dL (Figure 6C), which is typically expressed in oxidative stress scenarios in apoptotic cells [25]. This suggests a potential link between oxidative stress patterns in muscle cells and the development of NTIS.

In muscle cells, the endoplasmic reticulum (ER) is a highly specialized organelle responsible for muscle contraction. Protein synthesis and degradation are significant contributors to muscle wasting in the ICU. The ER requires chaperones to hold the normal protein folding to work correctly. Notably, the ER lumen enables protein modifications and maturation, especially the disulfide bond formation and glycosylation [25]. Among these chaperones are heat shock proteins, such as the glucose-regulated proteins 78 (GRP78) and 75 (GRP75). Around 30% of the proteins, including thiol-disulfide oxidoreductases, as the deiodinases, especially type 2, escape this control system and are defective. Considering this, we sought to analyze the GRPs 78 and 75 in the fiber muscle of ill patients.

**Figure 7:**
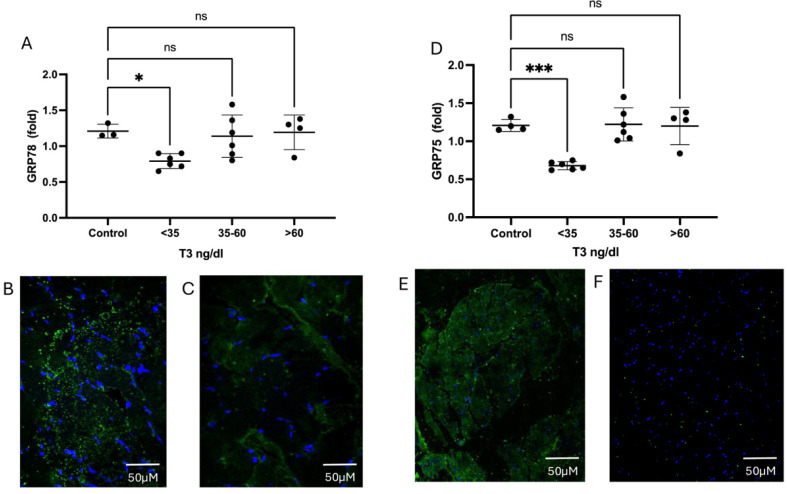
Expression and protein levels of GRP78 and GRP75 on muscle. (**A**) GRP78 mRNA levels. (**B**) GRP78 protein amount in controls and (**C**) protein of GRP78 in NTIS muscle. (**D**) GRP75 mRNA levels. (**E**) Protein amount in controls and (**F**) protein of GRP75 in NTIS sample. DAPI (blue), GRP78 and GRP75 (green). * *p* = 0.001, *** *p* < 0.0001. ns: Statistically not significant.

The results, as described in Figure 7, demonstrate that both GRP78 (Figure 7A, mRNA, Figure 7B, GRP78 protein amount in controls, and Figure 7C protein amount of GRP78 in NTIS muscle) and GRP75 (Figure 7D, mRNA, Figure 7E protein amount in controls and Figure 7F protein amount of GRP75 in NTIS) have their transcription and protein amount diminished.

## 3. Discussion

This prospective cohort study examined non-thyroidal illness syndrome (NTIS) and its clinical implications for critically ill patients, focusing on the molecular changes occurring in their muscle tissues. Potential muscle loss was assessed using ultrasound. Biopsy analysis revealed a reduction in sulfhydryl and glutathione (GSH) content. At the same time, carbonyl levels were increased in very low and low T3 groups, a trend also observed in serum samples. Notable findings included the expression and colocalization of deiodinases. DIO3 levels were significantly elevated in patients with the lowest serum T3 concentrations, predominantly in satellite cells, as evidenced by the colocalization of DIO3, MyoD+, and PAX7+ cells. In contrast, both DIO2 mRNA and D2 protein were increased among all critically ill patients, yet their distribution varied among the low T3 groups; specifically, the protein was found in greater concentrations within the stem cells of those with T3 levels below 35 ng/dL, while it was more concentrated around myocytes in the other groups. Additionally, we found that the inflammatory state of muscle tissue correlated with altered expression and levels of deiodinases and glucose transporters. Notably, the expression of the GLUT4 transporter was significantly reduced in patients with serum T3 levels below 35 ng/dL. Furthermore, Dio3 and GLUT4 appeared to colocalize, suggesting a link between NTIS and hyperglycemia. Conversely, IRS1 and IRS2 expression was diminished across all critically ill patients, irrespective of plasma T3 levels. T3 levels were undetectable in the muscle tissue of all patients with NTIS. Finally, both GRP78 and GRP75 chaperones were decreased in low T3 conditions, which may contribute to protein misfolding and partly explain the non-functional D2 protein. The elevated expression and protein levels of type 3 deiodinase in muscle satellite cells may represent a mechanism that inhibits their differentiation into mature muscle cells, thereby exacerbating lower T3 levels and increasing muscle loss in this population.

Non-thyroidal illness syndrome predicts outcomes in critically ill patients [16]. A meta-analysis of studies in ICU found relatively lower T3 concentrations among non-survivors, favoring the concept that thyroid dysfunction could indicate worse outcomes [16]. Our manuscript found a high prevalence (up to 88%) of NTIS compared to the literature despite the usual severity of predictive scores (SOFA and SAPS) assessed at the time of the patient’s ICU admission. We observed a precocity in NTIS development and its presence even in mild disease. This could explain the absence of difference in T3 levels among survivors and non-survivors. We also did not observe any differences in muscle thickness verified by ultrasound measurements between survivors and non-survivors. These findings are not consistent with other studies that used ultrasound as a method to verify muscle loss in ICU, which were able to detect mean losses of 10% in the first week of intensive care treatment, suggesting decreased protein synthesis since the patient’s admission, regardless of the nutritional load [13,26]. However, different factors could affect the sensitivity of point-of-care ultrasound, such as operators’ training and poor concordance among ultrasound performers. We minimized this bias by conducting a training program and a pilot study to analyze intra- and interobserver agreements. Notwithstanding, we verified a positive correlation between the variation in muscle thickness and weight gain during the first week of hospitalization, suggesting an influence of positive fluid balance on the cross-sectional area of the femoral quadriceps on the 7th day, which could partially explain these conflicting results. There was an inverse correlation between ICU length of stay and reduction of T3 levels in the first week of critical illness. In contrast, T3 levels of surviving patients increased, which did not happen among non-survivors. This suggests that NTIS resolution or attenuation precedes organic dysfunction recovery in those patients with better outcomes after ICU stay.

Although the role of stem cell dysfunction in critical illness-induced myopathy is not entirely understood, muscle repair mainly depends on them to constitute new myofibers in the repair process after injury [27,28]. Once the expression of types 2 and 3 deiodinases regulates the local T3 amount [29], the balance between them influences the proliferation and differentiation of stem cells [14]. Thus, a higher expression of DIO3 in stem cells is one of the mechanisms linked to the muscle regeneration impairment observed in critical illness, given that T3 potentiates stem cell differentiation [30]. According to previous data, decreased circulating T3 and elevated rT3 in NTIS patients are related to reduced D1 and D2 activity in the liver and skeletal muscle [20]. In contrast, D3 activity increases in the liver and skeletal muscle [20,31]. Our study found a putative non-functional D2 protein concentrated mainly in stem cells of low T3 patients and along the myocytes near normal to standard T3 levels.

Interestingly, while T3 was detected in the muscle of healthy controls, it was unmeasurable in cells in both deficient and low serum T3 level groups, suggesting an imbalance between D2 and D3 activity, in which inactivation of T4 into rT3 by D3 prevails, thus contributing to the setting of reduced TH stimuli to muscle cells. Our results add the potential misfolding of D2 protein through diminished chaperones in NTIS. There is growing evidence suggesting that T3 has its concentration decreased on a D3-dependent mechanism in the skeletal muscle of NTIS in animal models [32]. On the other hand, some data, also from animal models, show that the expression of deiodinases occurs in different types of cells and not at the same time during regeneration, as DIO2 was found to be expressed in Pax7+ cells later in the process, while DIO3 was detected in MyoD+ cells immediately post injury [33]. States of critical illness, like sepsis, affect quiescent muscle stem cells, impairing their development into mature cells by mitochondrial dysfunction, resulting in metabolic alterations. One study on mouse models assessed muscle regenerative capacity after induced sepsis, showing a marked decrease in satellite cell number as soon as 36 h after injury due to mitochondrial apoptosis. A septic environment also affected the long-term division potential in the remaining cells, suggesting that intrinsic and extrinsic mechanisms affect their division [29] and differentiation [34,35].

We also observed no significant difference in the expression of the MCT8 transporter in the muscle tissue regarding the serum T3 levels. This observation suggests that muscle dysfunction occurs at the intracellular level, with several alterations in most steps of cellular regulation, as detailed herein. Interestingly, some data show that thyroid hormone transporters are down-regulated in the skeletal muscle of patients with non-septic shock [36], which is not the case observed in the present study. Also, in concordance with our study, D2 protein did not differ between septic and healthy controls in previous findings [37]. Regarding our data on D2 and D3 expression in muscle cells, we can detail at least two scenarios in disease leading to muscle waste. The first T4 and T3 inactivation by D3 prevails over D2 activity since D2 plays a paramount role in converting TH from T4 to T3 in muscle tissue [38]. In the second scenario, together with augmented D3, there is a loss of D2 protein function that could occur through augmented oxidative stress and/or protein misfolding. Our findings cannot exclude any conditions contributing to critical illness muscle loss. It is also known that thyroid hormone promotes glucose transporter GLUT4 mRNA stability and translocation to the sarcolemma, leading to its upregulation and normal function. By this mechanism, THs enhance insulin-induced glucose uptake and muscle oxygen consumption, oxidative capacity, and resting metabolic activity through a Pgc1α-mediated increase in mitochondrial biogenesis, content, and function [14,39,40]. In the present study, we demonstrated a decreased expression of GLUT4 mRNA and protein in the acute phase of the disease in the group of patients with lower serum T3 concentration (<35 ng/dL). Interestingly, GLUT4 colocalizes at the MYOD+ cells containing D3, suggesting that the impairment of glucose metabolism in muscle is aggravated in severe NTIS scenarios.

The alterations in thyroid hormone metabolism concomitant with impaired mitochondrial homeostasis are caused by increased oxidative stress, as observed here. Consistent with our results in human muscle, the expression of Pgc1α (which is a regulator of ROS detoxification, mitochondrial biogenesis, metabolism, and muscle wasting) was decreased in septic models [41]. Indeed, this was observed in our group of patients with very low T3 that presented augmented BCL2 and CHOP expressions. Interestingly, it is known that CHOP expression inhibits myogenic differentiation by inhibition of the MYOD gene [42]. While BCL2 suggests that there might be an attempt to augment mitochondrial biogenesis, the augmented CHOP is a marker of ER stress. Moreover, CHOP inhibits myogenic differentiation, further contributing to muscle waste.

## 4. Materials and Methods

This prospective cohort was developed between October 2022 and September 2023, in which clinical, image, and histological information were obtained from critically ill patients. The institution’s Research Ethics Committee (CAAE: 60914122.2.0000.5327) approved the study protocol, and informed consent was obtained from all patients or their legal representatives.

### 4.1. Study Population

Two hundred and sixty-eight patients consecutively admitted to the intensive care unit of a tertiary hospital were screened. Critically ill patients were characterized by one or more life-threatening organ dysfunctions. They were eligible within the first 24 h of ICU admission if they had less than seven days of hospitalization. NTIS was characterized as low serum T3 with normal/low-normal TSH levels. Exclusion criteria were age under 18 years, known thyroid disease, irreversible clinical conditions or imminent death within the next 24 h, BMI > 35, chronic corticosteroid use, neurodegenerative diseases, or neurological patients with a high degree of sequelae (RANKIN greater than or equal to 4), pre-existing cachexia, and immediate postoperative period of elective surgery. Considering previous data on NTIS in sepsis [43], the prevalence of low T3 syndrome in critically ill patients, and the local mortality rate, the calculated sample size was 96 patients to detect a 20% difference in mortality between patients with low T3 syndrome and those without alterations, assuming a 95% confidence interval and a significance level of 90%. The control group was composed of 10 patients (5 males and 5 females) of matching age, without comorbidities, submitted to an elective surgery of hip arthroplasty. A muscle sample was obtained during surgery from the same muscle as the patients from the cohort.

### 4.2. Clinical, Laboratory, and Ultrasound Data

Clinical, demographic characteristics and severity scores were collected within the first 24 h of ICU admission. Venous blood for laboratory analysis was collected from all admitted patients. Ultrasound examination of the extensor muscles of the thigh using B-mode assesses the patient’s muscle thickness and potential loss during the first week of hospitalization. Four trained researchers performed ultrasound procedures. To minimize potential observation bias, the ultrasound operators were trained concurrently by an expert in the method, performing real-time measurement validation. Following this initial training period, a pilot study was conducted, which found no systematic disagreement among the examiners when compared to the senior intensivist, as assessed by the Bland–Altman test in most comparisons. Blood collection, clinic evaluation, and ultrasound were repeated on the seventh day of hospitalization for comparative purposes, outcome measurement, and the reassessment of clinical data such as weight and nutritional information during hospitalization. Serum concentrations of T3, tetraiodothyronine (T4), free-tetraiodothyronine (fT4), and thyroid stimulating hormone (TSH) were measured in the samples collected on admission and day 7 using electrochemiluminescence immunoassay, with 10 mL of blood obtained from enrolled patients. These analyses were conducted in the laboratory of biochemistry from Hospital de Clínicas de Porto Alegre, and the standard reference ranges used were as follows: FT4, 0.93–1.7 ng/dL; T4, 4.6–12 ng/dL; T3, 75–200 ng/dL; and TSH, 0.27–4.2 mU/L. Other analyses, such as HbA1C evaluation and measurement of plasma cytokines using multiplex assays (Human Magnetic Custom Luminex^®^ Kit, Invitrogen Life Technologies, Carlsbad, CA, USA), were also performed. Additional laboratory data presented in the results tables, including white blood cell count and C-reactive protein (CRP), were obtained through the analysis of medical records.

### 4.3. Muscle Biopsies

Surgical biopsies of skeletal muscle were conducted on the left vastus lateralis at the bedside, under aseptic technique and local anesthesia with 2% lidocaine without vasoconstrictor, with samples of at least 10 g, for evaluation of gene expression, biochemistry, and imaging. The specimens were rapidly isolated, washed in ice-cold 50 mM phosphate buffer saline, gently dried, immersed in RNALater solution (Thermo Fisher Scientific, Waltham, MA, USA), and stored at −80 °C until analyses. For histological studies, the samples were embedded in paraffin. The sections were then stained with hematoxylin–eosin (HE) to analyze the general morphology of the tissue. To further fiber analysis, skeletal muscle samples were embedded in an optimal cutting temperature compound (OCT Compound, Sigma-Aldrich, St. Louis, MO, USA) and frozen at −80 °C. Sections with a thickness of 5 μm were obtained for histological, immunohistochemical, and molecular tissue analyses.

### 4.4. Oxidative Stress Parameters

#### 4.4.1. Carbonyl Content

Carbonyl content was measured according to Zannata et al. [44]. The difference between the samples treated with 2,4-dinitrophenylhydrazine and treated with HCl (white) was used to calculate the carbonyl content determined at 370 nm. The data obtained were estimated by the millimolar absorption coefficient of hydrazine (e370 nm = 21.000000·M^−1^·cm^−1^), and the results were expressed in nmol carbonyl/mg of protein.

#### 4.4.2. Sulfhydryl Content

The sulfhydryl content, which represents the total thiol content of the tissue, was determined as described by Aksenov and Markesbery [45]. 5-thio-2-nitrobenzoic acid (TNB), derived from the reaction of thiols with 5,5′-dithiobis (2-nitrobenzoic acid), forms a yellow-colored derivative that is read in a spectrophotometer, measuring the absorbance at 412 nm. Results were expressed as nmol TNB/mg protein.

### 4.5. Antioxidant Defenses

#### 4.5.1. Reduced Glutathione Concentrations

The GSH parameter was measured as described by Browne and Armstrong [46]. Fluorescence was measured using excitation and emission wavelengths of 350 and 420 nm, respectively. The calibration curve was prepared with standard GSH (0.001–1 mM), and concentrations were calculated as GSH/mg protein nanomoles.

#### 4.5.2. Real-Time PCR

Total RNA was extracted from muscle tissue by the trizol method, cDNA was synthesized (SuperScript First-Strand Synthesis System for RT-PCR; Invitrogen), followed by real-time PCR with SYBR Green PCR Master Mix (Applied Biosystems, Waltham, MA, USA). Primers: DIO2: 5′-ACTTCCTGCTGGTCTACATTGATG-3′ and 5′-CTTCCTGGTTCTGGTGCTTCTTC-3′), DIO3: 5′-TCCAGAGCCAGCACATCCT-3′ and 5′-ACGTCGCGCTGGTACTTAGTG-3′ IRS 5′ CAACGGGCAGTTTGTCGAA 3′ and 5′ TGGTCGGGCAAACTTTCTG 3′, CHOP 5′CCAGCAGAGGTCACAAGCAC3 3′ and 5′ CGCACTGACCACTCTTGTTTC 3′, BCL2 GGCATCTGCACACCTGGAT and GGGCCATATAGTTCCACAAAGG, PGC1A 5′GGAGCAATAAAGCAAAGAGCA 3′ and 5′ GTGTGAGGAGGGTCATCGTT 3′ and YHWA internal control 5′ CCGCCAGGACAAACCAGTAT 3′, A and 5′ ACTTTTGGTACATTGTGGCTTCAA-3 (Applied Biosystems, Waltham, MA, USA) in the ABI Prism Vii7 Sequence Detection System (Applied Biosystems) assay. The r^2^ was more significant than 0.99, and the amplification efficiency varied between 80% and 100%. The delta-delta Ct method was used to calculate the fold change in gene expression compared with the control. Experiments were repeated at least three times.

### 4.6. Western Blot Analyses

Muscle samples were prepared as described by Fuziwara et al. [47]. Briefly, 30–50 μg of protein from each sample was fractionated by 8–12% SDS-PAGE and transferred to an Immobilon PVDF membrane (Millipore, Billerica, MA, USA). The following primary antibodies were used: anti-D3 (1:1000; Novus Biologicals, Englewood, CO, USA); and anti-β-actin (1:25,000; Sigma-Aldrich, St. Louis, MO, USA). Antigen–antibody complexes were visualized using HRP-conjugated secondary antibody and an enhanced chemiluminescence system (GE Healthcare, Pittsburgh, PA, USA). Expression was quantified using image densitometry with ImageJ 2 analysis software.

### 4.7. Immunofluorescence Studies

The immunofluorescence technique was modified according to Asakura et al. [48]. Briefly, muscle samples were fixed in 10% buffered formalin and placed on a silanized slide. Protein blocking was performed with 3% BSA for one hour at room temperature. Slides were permeabilized with 0.05% Tween 20 diluted in PBS. The incubation with the primary antibodies was overnight at four °C. The dilutions used were D3 (NBP1-05767, Novusbio, Englewood, CO, USA) 1:400 and D2 (Abcam, ab 77779, Cambridge, UK) with 1:200, GRP75 (MA1-094, Invitrogen-Thermo Fischer, Waltham, MA, USA) with 1:100, and GRP78 (MA5-27686, Invitrogen-Thermo Fischer, Waltham, MA, USA) with 1:100.

The secondary antibody was incubated for one hour and 30 min at room temperature with an anti-rabbit IgG secondary antibody (A11008, Invitrogen-Thermo Fischer, Waltham, MA, USA) at a 1:1000 dilution. The blades were mounted with a Fluoroshield mounting medium with Dapi (Abcam, ab104139, Cambridge, UK) and then analyzed using an Olympus FV1000 confocal microscope (Shinjuku, Japan), with a 40× objective.

### 4.8. Measurement of T3 in Tissue

We used a mass spectrometry (LCMS-8045, Shimadzu; Kyoto, Japan) analytical technique to measure T3 in cells. Tissue was homogenized with 500 µL of ice-cold extraction solvent (methanol/water; 80:20). The pellet was immediately transferred to liquid nitrogen for 10 min and then thawed in ice for 10 min. The freezing and thawing cycle was repeated twice for complete cell rupture. After the last thawing, the pellet was centrifuged for 5 min at 12,000 rpm, and the supernatant was collected for immediate measurements.

### 4.9. RNAscope In Situ Hybridization

In situ hybridization for D2 and D3, as well as Pax7, MyoD, and PDGFA, was performed on representative selected sections using the RNAscope technique (Advanced Cell Diagnostics, BioTechne Corporation, Newark, CA, USA) following the manufacturer’s suggested procedures. This approach determined the spatial localization of Dio2 and Dio3 expression and associated genes. We used the RNAscope DIO2, DIO3, PDGFA, PAX7, and MYOD probes (catalog numbers 562211, 562861, 406721, 546691, and 562721, respectively) and the Akoya Detection Kit (Marlborough, MA, USA) to obtain the fluorescence results, as indicated by the manufacturer. As negative and positive controls, we used the material supplied by the manufacturer, which had the expected negative or positive results. Tissue sections were mounted with DAPI Fluoromount-G (Catalog # 0100-20, Southern Biotech, Birmingham, AL, USA). Samples were imaged using a Nikon H600L microscope with a 40× objective (Nikon, Tokyo, Japan). The images shown here are illustrative of the complete sections analyzed. H&E stains were performed to verify the quality of the sample before each experiment.

### 4.10. Statistical Analysis

The database and statistical analysis were conducted using the SPSS software 30.0. A statistically significant threshold was set at *p* < 0.05. Descriptive data were presented as the mean and standard deviation or the median and interquartile range, depending on the normality distribution, for continuous variables and percentages for categorical variables. Measure differences were assessed using the Mann–Whitney U test for non-parametric variables expressed in medians and interquartile ranges and the *t*-test for variables with parametric distribution. Correlations between variables were determined using the Pearson or Spearman test, depending on the sample distribution. ANOVA was performed to compare means across three or more independent groups.

## 5. Conclusions

Our results add to the current understanding of how multiple harmful pathological processes in patients with critical illnesses, such as inflammation and energetic imbalance, mediate muscular molecular alterations and play an essential role in adverse outcomes in this population. In conclusion, the present study provides new evidence that altered thyroid hormone metabolism plays a vital role in the dysfunction of stem and muscle cells since there is a whole new described crosstalk among deiodinases, glucose transporters, mitochondrial function, and ER stress that further explains the complex mechanism of critical illness-induced myopathy.

## Figures and Tables

**Table 1:** Demographic and clinical parameters of the patients.

	Total (n = 96)	Survivors (n = 68)	Non-Survivors (n = 28)
**Demographic Characteristics on Day One—Means (±sd)**			
Age (y)	59.65	59.3 ± 13.6	60.5 ± 16.1
Male (%)	56%	56.5	53.6
Sepsis or septic shock (%)	67	69.3	64.6
**Predictive Scores on Admission—Means (±sd)**			
SAPS	67.2 ± 17.3	65.7 ± 16.8	71.1 ± 18.4
SOFA	7.3 ± 4	7.36 ± 4	7.47 ± 4
**Laboratory Findings on admission—Median (IQR)**			
Reactive C-protein	168 (77–281)	139 (51–300)	187 (90–276)
White blood cell	12,140 (7860–17,155)	11,530 (7660–16,680)	13,185 (9310–19,620)
**Thyroid Hormones—Median (IQR)**			
NTIS (%)	88.7	88.4	89.3
TSH	0.85 (0.37–1.61)	0.87 (0.40–1.43)	0.82 (0.27–2.35)
Total T4	5.93 (4.53–6.99)	5.96 (4.64–7.00)	5.65 (3.51–6.91)
Free T4	0.89 (0.75–1.06)	0.93 (0.79–1.07)	0.83 (0.72–0.97)
T3	34.2 (30.0–48.2)	32.5 (30.0–46.8)	36.0 (30.0–53.1)
**Ultrasound Measurements—Means (±sd)**			
Quadriceps thickness (cm)	2.22 ± 0.78	2.17 ± 0.8	2.33 ± 0.74

**Table 2:** Laboratory, caloric, and protein intake, and ultrasound on day 7.

Clinical Findings on Day 7				
Variable	Total (n = 59)	Survivors (n = 44)	Non-Survivors (n = 15)	
Median T3 (ng/dL) *	42.4 (30.0–53.0)	45.0 (31.3–56.5)	39.5 (30.0–47.8)	*p* = 0.085
Mean quadriceps thickness (cm) *	1.82 ± 0.63	1.85 ± 0.52	2.1 ± 0.8	*p* = 0.165
Proposed caloric intake achieved % **	75	79	64	*p* = 0.296
Protein intake achieved (g/kg/d)	1.50 (1.24–1.75)	1.53 (1.31–1.75)	1.50 (0.72–1.71)	*p* = 0.333

* Mann–Whitney, ** q-square.

## Data Availability

The data presented in this study are available on request from the corresponding author. The data are not publicly available because some data is still unpublished.

## References

[1] Salvatore D., Simonides W.S., Dentice M., Zavacki A.M., Larsen P.R. (2014). Thyroid hormones and skeletal muscle—New insights and potential implications. Nat. Rev. Endocrinol..

[2] Kelmenson D.A., Held N., Allen R.R., Quan D., Burnham E.L., Clark B.J., Ho P.M., Kiser T.H., Vandivier R.W., Moss M. (2017). Outcomes of ICU Patients with a Discharge Diagnosis of Critical Illness Polyneuromyopathy: A Propensity-Matched Analysis. Crit. Care Med..

[3] Latronico N., Herridge M., Hopkins R.O., Angus D., Hart N., Hermans G., Iwashyna T., Arabi Y., Citerio G., Ely E.W. (2017). The ICM research agenda on intensive care unit-acquired weakness. Intensive Care Med..

[4] Dres M., Jung B., Molinari N., Manna F., Dubé B.-P., Chanques G., Similowski T., Jaber S., Demoule A. (2019). Respective contribution of intensive care unit-acquired limb muscle and severe diaphragm weakness on weaning outcome and mortality: A post hoc analysis of two cohorts. Crit. Care Lond. Engl..

[5] Manning E.M., Shenkin A. (1995). Nutritional assessment in the critically ill. Crit. Care Clin..

[6] Chen J., Huang M. (2024). Intensive casre unit-acquired weakness: Recent insights. J. Intensive Med..

[7] Chi-Fishman G., Hicks J.E., Cintas H.M., Sonies B.C., Gerber L.H. (2004). Ultrasound imaging distinguishes between normal and weak muscle. Arch. Phys. Med. Rehabil..

[8] Freilich R.J., Kirsner R.L., Byrne E. (1995). Isometric strength and thickness relationships in human quadriceps muscle. Neuromuscul. Disord. NMD.

[9] Miyatani M., Kanehisa H., Kuno S., Nishijima T., Fukunaga T. (2002). Validity of ultrasonograph muscle thickness measurements for estimating muscle volume of knee extensors in humans. Eur. J. Appl. Physiol..

[10] Paris M.T., Mourtzakis M., Day A., Leung R., Watharkar S., Kozar R., Earthman C., Kuchnia A., Dhaliwal R., Moisey L. (2017). Validation of Bedside Ultrasound of Muscle Layer Thickness of the Quadriceps in the Critically Ill Patient (VALIDUM Study). JPEN J. Parenter. Enteral. Nutr..

[11] Agyapong-Badu S., Warner M., Samuel D., Narici M., Cooper C., Stokes M. (2014). Anterior thigh composition measured using ultrasound imaging to quantify relative thickness of muscle and non-contractile tissue: A potential biomarker for musculoskeletal health. Physiol. Meas..

[12] Puthucheary Z.A., Rawal J., McPhail M., Connolly B., Ratnayake G., Chan P., Hopkinson N.S., Phadke R., Dew T., Sidhu P.S. (2013). Acute skeletal muscle wasting in critical illness. JAMA.

[13] Dupont A.C., Sauerbrei E.E., Fenton P.V., Shragge P.C., Loeb G.E., Richmond F.J. (2001). Real-time sonography to estimate muscle thickness: Comparison with MRI and CT. J. Clin. Ultrasound JCU.

[14] Bloise F.F., Cordeiro A., Ortiga-Carvalho T.M. (2018). Role of thyroid hormone in skeletal muscle physiology. J. Endocrinol..

[15] Mebis L., Paletta D., Debaveye Y., Ellger B., Langouche L., D’Hoore A., Darras V.M., Visser T.J., Berghe G.V.D. (2009). Expression of thyroid hormone transporters during critical illness. Eur. J. Endocrinol..

[16] Brunetto E.L., Teixeira S.D.S., Giannocco G., Machado U.F., Nunes M.T. (2012). T3 rapidly increases SLC2A4 gene expression and GLUT4 trafficking to the plasma membrane in skeletal muscle of rat and improves glucose homeostasis. Thyroid. Off J. Am. Thyroid. Assoc..

[17] Zanquetta M.M., Alves-Wagner A.B., Mori R.C., Campello R.S., Machado U.F. (2014). Recovery of insulin sensitivity and Slc2a4 mRNA expression depend on T3 hormone during refeeding. Metabolism.

[18] Weinstein S.P., O’Boyle E., Haber R.S. (1994). Thyroid hormone increases basal and insulin-stimulated glucose transport in skeletal muscle: The role of GLUT4 glucose transporter expression. Diabetes.

[19] Vidart J., Jaskulski P., Kunzler A.L., Marschner R.A., Ferreira de Azeredo da Silva A., Wajner S.M. (2022). Non-thyroidal illness syndrome predicts outcome in adult critically ill patients: A systematic review and meta-analysis. Endocr. Connect..

[20] Maldonado L.S., Murata G.H., Hershman J.M., Braunstein G.D. (1992). Do thyroid function tests independently predict survival in the critically ill?. Thyroid. Off. J. Am. Thyroid. Assoc..

[21] Sagliocchi S., Cicatiello A.G., Di Cicco E., Ambrosio R., Miro C., Di Girolamo D., Nappi A., Mancino G., De Stefano M.A., Luongo C. (2019). The thyroid hormone activating enzyme, type 2 deiodinase, induces myogenic differentiation by regulating mitochondrial metabolism and reducing oxidative stress. Redox Biol..

[22] Gereben B., Zavacki A.M., Ribich S., Kim B.W., Huang S.A., Simonides W.S., Zeold A., Bianco A.C. (2008). Cellular and Molecular Basis of Deiodinase-Regulated Thyroid Hormone Signaling1. Endocr. Rev..

[23] Peeters R.P., van der Geyten S., Wouters P.J., Darras V.M., van Toor H., Kaptein E., Visser T.J., Berghe G.V.D. (2005). Tissue thyroid hormone levels in critical illness. J. Clin. Endocrinol. Metab..

[24] da Silva Rosa S.C., Nayak N., Caymo A.M., Gordon J.W. (2020). Mechanisms of muscle insulin resistance and the cross-talk with liver and adipose tissue. Physiol. Rep..

[25] Malhotra J.D., Kaufman R.J. (2007). The endoplasmic reticulum and the unfolded protein response. Seminars in Cell & Developmental Biology.

[26] Parry S.M., El-Ansary D., Cartwright M.S., Sarwal A., Berney S., Koopman R., Annoni R., Puthucheary Z., Gordon I.R., Morris P.E. (2015). Ultrasonography in the intensive care setting can be used to detect changes in the quality and quantity of muscle and is related to muscle strength and function. J. Crit. Care..

[27] Lepper C., Partridge T.A., Fan C.M. (2011). An absolute requirement for Pax7-positive satellite cells in acute injury-induced skeletal muscle regeneration. Dev. Camb. Engl..

[28] Murphy M.M., Lawson J.A., Mathew S.J., Hutcheson D.A., Kardon G. (2011). Satellite cells, connective tissue fibroblasts and their interactions are crucial for muscle regeneration. Dev. Camb. Engl..

[29] Lassar A.B., Paterson B.M., Weintraub H. (1986). Transfection of a DNA locus that mediates the conversion of 10T1/2 fibroblasts to myoblasts. Cell.

[30] Wosczyna M.N., Rando T.A. (2018). A Muscle Stem Cell Support Group: Coordinated Cellular Responses in Muscle Regeneration. Dev. Cell.

[31] Peeters R.P., Wouters P.J., Kaptein E., van Toor H., Visser T.J., Van den Berghe G. (2003). Reduced activation and increased inactivation of thyroid hormone in tissues of critically ill patients. J. Clin. Endocrinol. Metab..

[32] Lehnen T.E., Marschner R., Dias F., Maia A.L., Wajner S.M. (2020). Oxidative remote induction of type 3 deiodinase impacts nonthyroidal illness syndrome. J. Endocrinol..

[33] Ogawa-Wong A., Carmody C., Le K., Marschner R.A., Larsen P.R., Zavacki A.M., Wajner S.M. (2022). Modulation of Deiodinase Types 2 and 3 during Skeletal Muscle Regeneration. Metabolites.

[34] Andrés V., Walsh K. (1996). Myogenin expression, cell cycle withdrawal, and phenotypic differentiation are temporally separable events that precede cell fusion upon myogenesis. J. Cell Biol..

[35] Rocheteau P., Chatre L., Briand D., Mebarki M., Jouvion G., Bardon J., Crochemore C., Serrani P., Lecci P.P., Latil M. (2015). Sepsis induces long-term metabolic and mitochondrial muscle stem cell dysfunction amenable by mesenchymal stem cell therapy. Nat. Commun..

[36] Lado-Abeal J., Romero A., Castro-Piedras I., Rodriguez-Perez A., Alvarez-Escudero J. (2010). Thyroid hormone receptors are down-regulated in skeletal muscle of patients with non-thyroidal illness syndrome secondary to non-septic shock. Eur. J. Endocrinol..

[37] Rodriguez-Perez A., Palos-Paz F., Kaptein E., Visser T.J., Dominguez-Gerpe L., Alvarez-Escudero J., Lado-Abeal J. (2008). Identification of molecular mechanisms related to nonthyroidal illness syndrome in skeletal muscle and adipose tissue from patients with septic shock. Clin. Endocrinol..

[38] Dentice M., Marsili A., Zavacki A., Larsen P.R., Salvatore D. (2013). The deiodinases and the control of intracellular thyroid hormone signaling during cellular differentiation. Biochim. Biophys. Acta.

[39] Mishra P., Varuzhanyan G., Pham A.H., Chan D.C. (2015). Mitochondrial Dynamics is a Distinguishing Feature of Skeletal Muscle Fiber Types and Regulates Organellar Compartmentalization. Cell Metab..

[40] Schnyder S., Handschin C. (2015). Skeletal muscle as an endocrine organ: PGC-1α, myokines and exercise. Bone.

[41] Rius-Pérez S., Torres-Cuevas I., Millán I., Ortega Á.L., Pérez S. (2020). PGC-1α, Inflammation, and Oxidative Stress: An Integrative View in Metabolism. Oxid. Med. Cell Longev..

[42] Kny M., Fielitz J. (2022). Hidden Agenda—The Involvement of Endoplasmic Reticulum Stress and Unfolded Protein Response in Inflammation-Induced Muscle Wasting. Front. Immunol..

[43] Vidart J., Axelrud L., Braun A.C., Marschner R.A., Wajner S.M. (2023). Relationship among low T3 levels, type 3 deiodinase, oxidative stress, and mortality in sepsis and septic shock: Defining patient outcomes. Int. J. Mol. Sci..

[44] Zanatta Â., Viegas C.M., Tonin A.M., Busanello E.N.B., Grings M., Moura A.P., Leipnitz G., Wajner M. (2013). Disturbance of redox homeostasis by ornithine and homocitrulline in rat cerebellum: A possible mechanism of cerebellar dysfunction in HHH syndrome. Life Sci..

[45] Aksenov M.Y., Markesbery W.R. (2001). Changes in thiol content and expression of glutathione redox system genes in the hippocampus and cerebellum in Alzheimer’s disease. Neurosci. Lett..

[46] Browne R.W., Armstrong D. (1998). Reduced glutathione and glutathione disulfide. Free. Radic. Antioxid. Protoc..

[47] Fuziwara C.S., Kimura E.T. (2014). High iodine blocks a Notch/miR-19 loop activated by the BRAF(V600E) oncoprotein and restores the response to TGFβ in thyroid follicular cells. Thyroid. Off J. Am. Thyroid. Assoc..

[48] Asakura A., Kikyo N. (2022). Immunofluorescence analysis of myogenic differentiation. Methods Cell Biol..

